# High Immobilization Efficiency of Basic Protein within Heparin-Immobilized Calcium Phosphate Nanoparticles

**DOI:** 10.3390/ijms231911530

**Published:** 2022-09-29

**Authors:** Maki Nakamura, Wakako Bunryo, Aiko Narazaki, Ayako Oyane

**Affiliations:** 1 Nanomaterials Research Institute, National Institute of Advanced Industrial Science and Technology (AIST), Central 5, 1-1-1 Higashi, Tsukuba 305-8565, Ibaraki, Japan; 2 Research Institute for Advanced Electronics and Photonics, National Institute of Advanced Industrial Science and Technology (AIST), Central 2, 1-1-1 Umezono, Tsukuba 305-8568, Ibaraki, Japan

**Keywords:** calcium phosphate, nanoparticle, immobilization, cytochrome C, basic protein

## Abstract

Previously, we achieved one-pot fabrication of heparin-immobilized calcium phosphate (CaP) nanoparticles with high dispersibility by a precipitation process in a highly supersaturated reaction solution. In this study, we revealed that the heparin-immobilized CaP nanoparticles have a greater co-immobilizing capacity for basic proteins than for acidic proteins. In this process, heparin acted as not only a particle-dispersing agent but also as an immobilizing agent for basic proteins; it remarkably (approximately three-fold) improved the immobilization efficiency of cytochrome C (a model basic protein) within the CaP nanoparticles. The content of cytochrome C immobilized within the nanoparticles was increased with an increase in cytochrome C concentration in the reaction solution and by aging the nanoparticles. The obtained nanoparticles were dispersed well in water owing to their large negative zeta potentials derived from heparin, irrespective of the content of cytochrome C. Similar results were obtained also for another basic protein, lysozyme, but not for an acidic protein, albumin; the immobilization efficiency of albumin within the nanoparticles was decreased by heparin. These findings provide new insights into the co-immobilization strategy of proteins within heparin-immobilized CaP nanoparticles and will be useful in the design and fabrication of nanocarriers for protein delivery applications.

## 1. Introduction

Biomolecules (proteins, nucleic acids, etc.), produced by living organisms are attractive as therapeutic agents for advanced therapies including cell therapy, gene therapy, and molecular-targeted therapy. However, biomolecules are generally unstable in the body and exhibit biological activity in a dose-dependent matter. Thus, suitable delivery carriers of biomolecules are required to control their physiological dynamics and local concentration at a target site within the body. Calcium phosphate (CaP) nanoparticles (defined here as particles with a diameter of 1–1000 nm) have a high potential as delivery carriers of biomolecules [1,2,3,4]. This is because certain CaP compounds, chemically similar to human biominerals, show a high affinity for various biomolecules and are biodegradable into serum ions (calcium and phosphate ions) with minimal toxicity.

There are many methods for fabricating CaP nanoparticles, including chemical, physical, and physicochemical processes [5,6,7]. Among them, a chemical precipitation process, based on homogeneous CaP nucleation in a highly supersaturated CaP solution, is useful for fabricating CaP nanoparticles to immobilize biomolecules. This process is carried out under nearly physiological conditions (near-neutral pH, near-isotonic salt solutions, at or below body temperature) and thus is unlikely to cause denaturation of the biomolecules. However, the precipitation process makes it difficult to control the size and dispersibility of the nanoparticles. To deal with this issue, synthetic surfactants have often been used despite their safety concerns.

In recent years, certain types of biomolecules have been applied as dispersants in place of synthetic surfactants in the precipitation process of CaP-based nanoparticles [8]. For example, nucleic acids [9,10], proteins [11,12], and polysaccharides [13,14,15,16,17] have been immobilized on or within CaP nanoparticles, thereby allowing their dispersion even in the absence of synthetic surfactants. Among these biomolecules, heparin is of particular interest, since it is the oldest and most commonly used antithrombotic drug and has a high affinity with CaPs. Heparin is a polysaccharide having negatively charged sulfo and carboxyl groups and has been reported to act as a dispersant when immobilized within CaP-based nanoparticles [14,16,17]. We recently achieved one-pot fabrication of CaP nanoparticles co-immobilizing heparin and iron oxide (IO) nanocrystals (magnetic resonance imaging contrast agents) via coprecipitation in a highly supersaturated CaP solution supplemented with heparin and IO nanocrystals [16,17]. The resulting nanoparticles had hydrodynamic diameters of a few hundred nanometers and dispersed well in water compared to the heparin-free nanoparticles (nanoparticles immobilizing IO nanocrystals only) due to their large negative zeta potential derived from heparin. If other biofunctional reagents besides IO nanocrystals can also be co-immobilized within heparin-immobilized CaP nanoparticles, the resulting nanoparticles may exert a variety of functions depending on the co-immobilized reagents. However, there are few reports on the co-immobilization of biofunctional reagents within heparin-immobilized CaP nanoparticles.

Herein, we developed a co-immobilization strategy for biomolecules, especially proteins, within heparin-immobilized CaP nanoparticles. Negatively charged heparin has a high affinity with basic proteins through electrostatic interactions [18,19]. Thus, we hypothesized that basic proteins should be co-immobilized within heparin-immobilized CaP nanoparticles with higher efficiency than acidic proteins by a precipitation process. Some basic proteins, such as cytokines (e.g., fibroblast growth factor-2, bone morphogenetic protein-2, and interferon γ) and antibodies (e.g., trastuzumab and denosumab), are clinically used as biological drugs. Hence, it is worth investigating delivery carriers for basic proteins for advanced therapy. In this study, among basic proteins, cytochrome C (isoelectric point (PI) = 10.1 [19]) was selected as a model protein for co-immobilization. To verify our hypothesis, another basic protein, lysozyme (PI = 10.7 [19]), and an acidic protein, albumin (PI = 4.7 [12]) were also tested for comparison.

The precipitation process was performed in a highly supersaturated CaP solution: a reaction solution containing calcium ions, phosphate ions, heparin, and the selected protein (cytochrome C, lysozyme, or albumin). The reaction solution was mixed at room temperature for 1 min, and the nanoparticles were collected just after mixing (without aging) or after subsequent aging for 24 h. The resultant nanoparticles were analyzed for their physicochemical properties and immobilization efficiency of the protein, i.e., the percentage of the protein content in the nanoparticles over the total amount of the protein added to the reaction solution. The results are discussed in terms of the combination of biomolecules for co-immobilization and the reaction conditions.

## 2. Results

### 2.1. Morphological Analysis of Products

**Figure 1:**
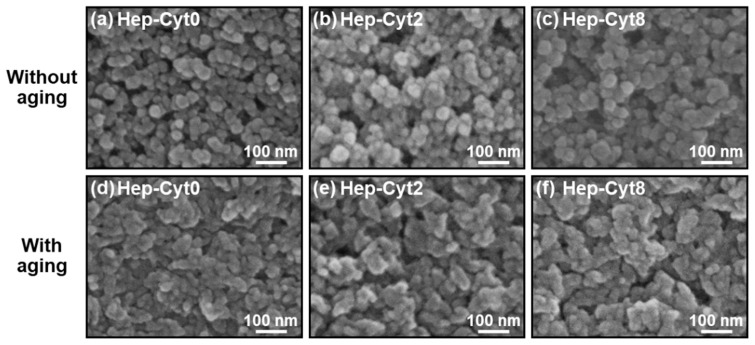
SEM images of the products: **Hep-Cyt0**, **Hep-Cyt2**, and **Hep-Cyt8** with (**d**–**f**) and without (**a**–**c**) aging.

**Figure 2:**
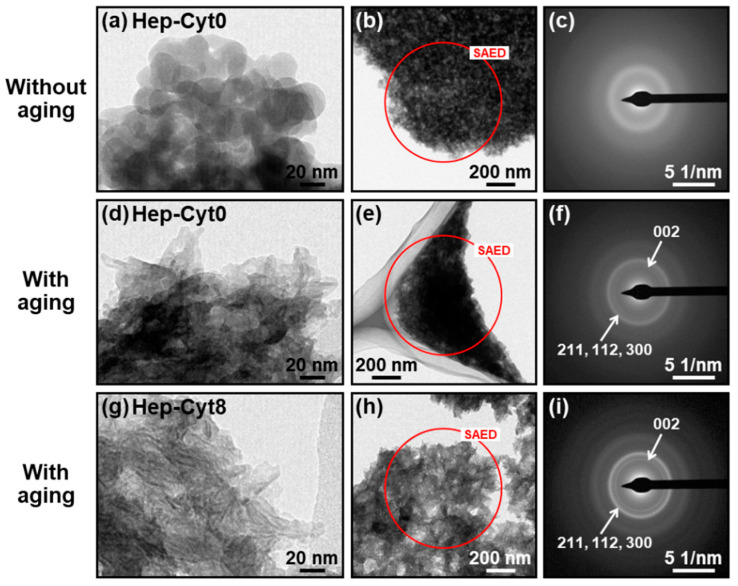
TEM images (**a**,**b**,**d**,**e**,**g**,**h**) and SAED (**c**,**f**,**i**) patterns of the products: **Hep-Cyt0** with (**d**–**f**) and without (**a**–**c**) aging, and **Hep-Cyt8** with aging (**g**–**i**). SAED patterns were taken from the red circular area in (**b**,**e**,**h**).

In the reaction solution containing heparin and cytochrome C, spherical nanoparticles were formed just after mixing and deformed by subsequent aging for 24 h, irrespective of the concentration of cytochrome C. First, we prepared six types of reaction solutions containing heparin (fixed concentration) and various concentrations of cytochrome C by mixing three source solutions A, B, and C (for details, see Section 4.1). The products obtained with and without aging are represented as **Hep-Cyt*n*** (*n* = 0, 1, 2, 3, 5, and 8) where *n* is the cytochrome C concentration (mg/mL) of solution C. As shown in the scanning electron microscope (SEM) images, **Hep-Cyt*n*** (*n* = 0, 1, 2, 3, 5, and 8) without aging were composed of nanoparticles with a primary particle diameter of less than 50 nm with an approximately spherical shape (Figure 1a–c and Figure S1a–c). After aging, these nanoparticles obtained an irregular shape (Figure 1d–f and Figure S1d–f). In transmission electron microscope (TEM) images, **Hep-Cyt0** without aging was comprised of approximately spherical nanoparticles with a primary diameter of about 20–30 nm (Figure 2a), whereas **Hep-Cyt0** and **Hep-Cyt8** with aging showed irregularly shaped nanoparticles with indistinct boundaries (Figure 2d,g). The results of selected area electron diffraction (SAED) analysis (Figure 2b,c,e,f,h,i) are described in Section 2.4.

### 2.2. Immobilization Efficiencies of Cytochrome C within Nanoparticles

**Figure 3:**
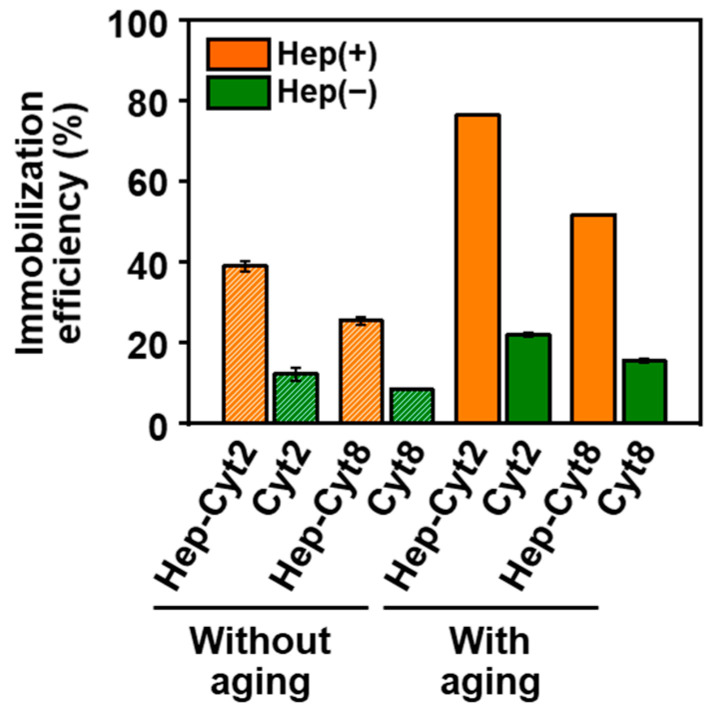
Immobilization efficiencies of cytochrome C in the nanoparticles: **Hep-Cyt2**, **Cyt2**, **Hep-Cyt8**, and **Cyt8** with and without aging (average ± standard error, N (number of batches) = 2 for **Cyt2** and **Cyt8** with aging, N = 3 for others).

The addition of heparin to the reaction solution improved the immobilization efficiency of cytochrome C within the nanoparticles. Here, heparin-free nanoparticles, represented as **Cyt*n*** (*n* = 2 and 8), were prepared from cytochrome C-containing reaction solutions (without heparin) with and without aging, and compared with **Hep-Cyt*n*** (*n* = 2 and 8). The nanoparticles immobilized different contents of cytochrome C depending on the reaction conditions. Before aging, the immobilization efficiencies of cytochrome C for **Hep-Cyt2** and **Hep-Cyt8** were 3.2 and 3.0 times larger than those for **Cyt2** and **Cyt8**, respectively (Figure 3). There were significant differences in the immobilization efficiencies of cytochrome C between **Hep-Cyt2** and **Cyt2** (*p* < 0.001, *t*-test), and between **Hep-Cyt8** and **Cyt8** (*p* < 0.01, *t*-test). This result indicated that heparin added to the reaction solution enhanced the immobilization of cytochrome C within the nanoparticles in the initial mixing process (before subsequent aging). For all the nanoparticles listed above, the immobilization efficiency of cytochrome C increased after aging (Figure 3), suggesting that more cytochrome C was immobilized within the nanoparticles during aging. In **Hep-Cyt*n*** (*n* = 1, 2, 3, 5, and 8) with and without aging, the content of cytochrome C increased with increasing cytochrome C concentration in the reaction solution (Figure S2a). This indicated that more cytochrome C was immobilized within the CaP nanoparticles in the reaction solution with higher cytochrome C concentration. On the other hand, the immobilization efficiency of cytochrome C decreased with increasing cytochrome C concentration in the reaction solution (Figure S2b). This might be because of the limited number of immobilization sites on the CaP nanoparticles with respect to the excess amount of cytochrome C, leading to the increased rate of cytochrome C that remained in the reaction solution.

### 2.3. Chemical Analysis of Nanoparticles

**Figure 4:**
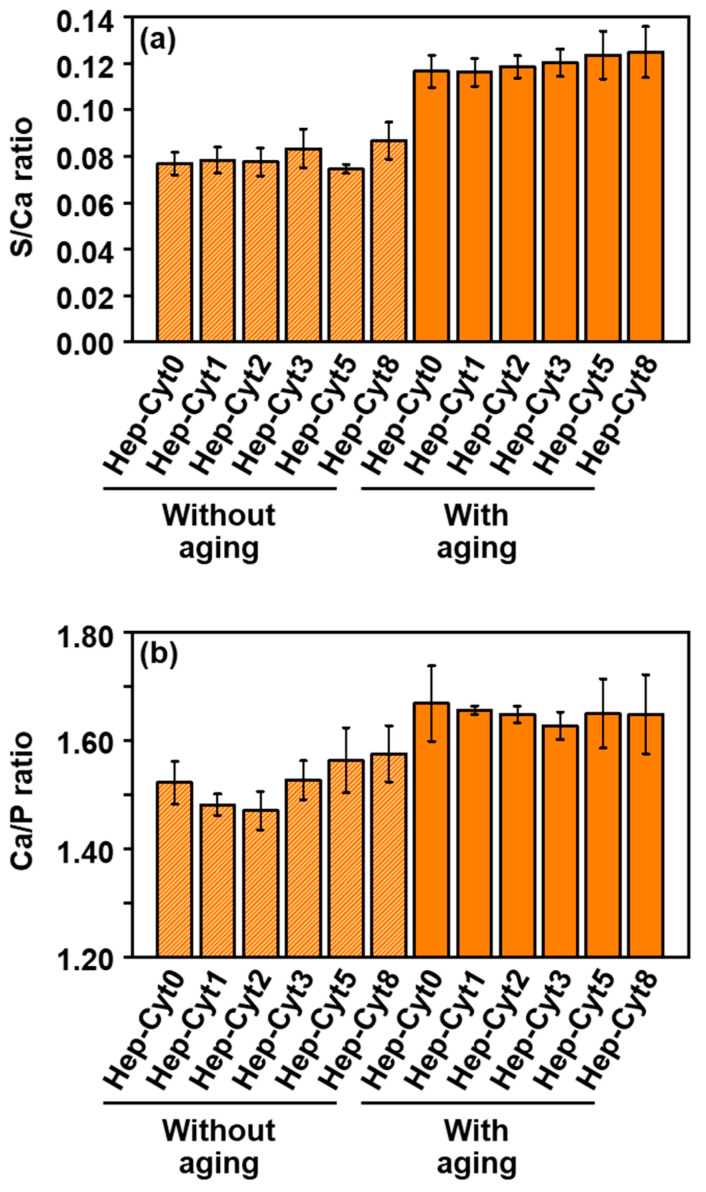
Elemental ratios of S/Ca (**a**) and Ca/P (**b**) of the nanoparticles: **Hep-Cyt0**, **Hep-Cyt1**, **Hep-Cyt2**, **Hep-Cyt3**, **Hep-Cyt5**, and **Hep-Cyt8** with and without aging (average ± standard error, N = 2).

The nanoparticles obtained from the heparin-containing reaction solutions were composed of CaP and immobilized heparin, irrespective of their cytochrome C content. In the energy dispersive X-ray spectroscopy (EDX) results for **Hep-Cyt*n*** (*n* = 0, 2, and 8) with and without aging, strong peaks of O, P, and Ca were detected, indicating that all these nanoparticles mainly consisted of CaP (Figure S3). In addition to these peaks, a small peak of S was also detected. The observed S peak was attributed mostly to heparin rather than cytochrome C (both of which possess S in their chemical structures) immobilized within the nanoparticles. This is because the content of S derived from cytochrome C was only less than 10% of the total content of S in these nanoparticles (estimated from the inductively coupled plasma-optical emission spectrometer (ICP-OES) results described below). The contents of Ca, P, and S in the nanoparticles were determined using ICP-OES (Figure S4). The elemental ratios of S/Ca calculated from the S and Ca contents were similar among **Hep-Cyt*n*** (*n* = 0, 1, 2, 3, 5, and 8) both with and without aging (Figure 4a). The results showed that the heparin content in the nanoparticles was hardly influenced by the concentration of cytochrome C in the reaction solution. The content of heparin in the nanoparticles increased remarkably after aging (about 40–60% increase), whereas the changes in Ca and P contents were less than 15% (Figure S4a,b). Consequently, **Hep-Cyt*n*** (*n* = 0, 1, 2, 3, 5, and 8) with aging had the higher elemental ratios of S/Ca (ca. 0.12) than those without aging (ca. 0.08) (Figure 4a). These results indicate that CaP nanoparticles were enriched with heparin during aging via further heparin immobilization while CaP growth was decelerated in the reaction solution.

The Ca/P elemental ratio of the heparin-immobilized CaP nanoparticles was increased after aging. The Ca/P elemental ratios of **Hep-Cyt*n*** (*n* = 0, 1, 2, 3, 5, and 8) without aging were lower than 1.60 (from 1.47 to 1.58), whereas those with aging were higher than 1.60 (from 1.63 to 1.67) and closer to that of stoichiometric hydroxyapatite (1.67) (Figure 4b). This might be due to the crystalline structural change in the CaP phase in the nanoparticles during aging as described in the following section.

### 2.4. Electron Diffraction Analysis of Nanoparticles

The heparin-immobilized CaP nanoparticles formed in the heparin-containing reaction solution just after mixing were amorphous and underwent crystallization in the subsequent aging process. The SAED pattern of **Hep-Cyt0** without aging showed neither sharp rings nor spots and provided only a broad ring ascribed to amorphous CaP (Figure 2c). In contrast, **Hep-Cyt0** with aging showed two concentric diffraction rings ascribed to crystalline apatite (a kind of CaP crystal) (Figure 2f). Similar diffraction rings were also obtained from **Hep-Cyt8** with aging (Figure 2i), suggesting that the aged CaP nanoparticles immobilizing heparin and cytochrome C were comprised of crystalline apatite.

### 2.5. Size Distribution and Zeta Potential of Nanoparticles

**Figure 5:**
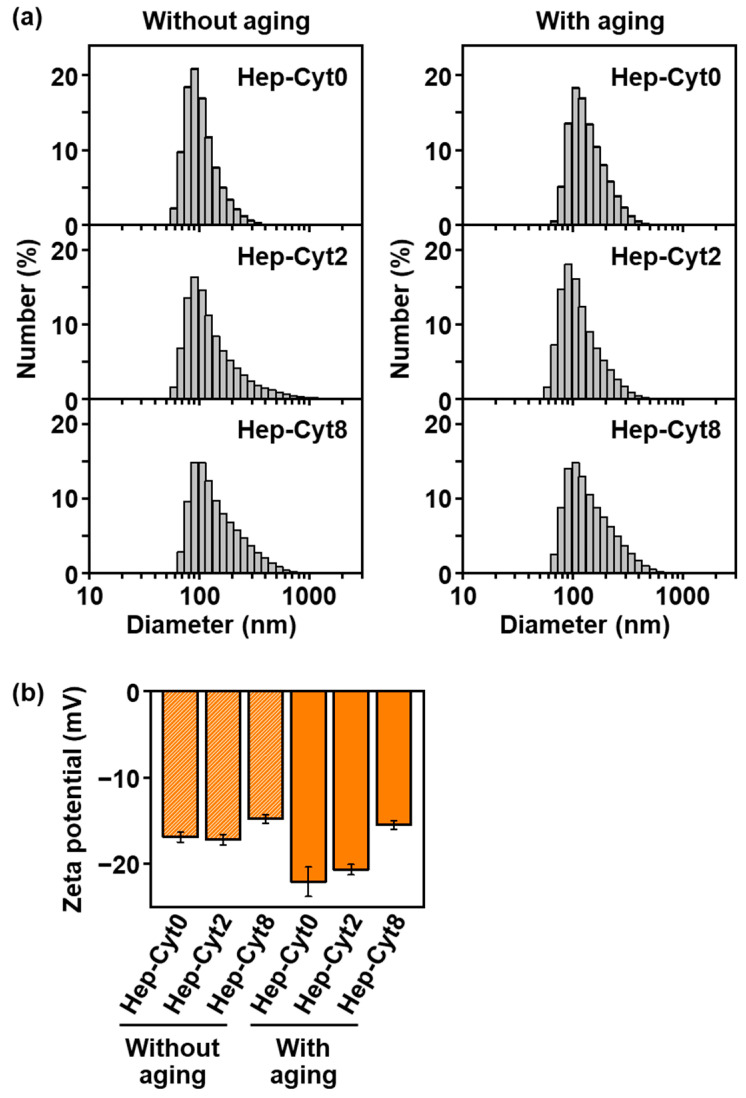
DLS histograms of the number distributions (**a**) and zeta potentials (**b**) of the nanoparticles: **Hep-Cyt0**, **Hep-Cyt2**, and **Hep-Cyt8** with and without aging. The nanoparticles were dispersed in water.

With or without aging, the heparin-immobilized CaP nanoparticles were dispersed in water, although the heparin-free nanoparticles were not, regardless of their cytochrome C content. The nanoparticles obtained from the heparin-containing reaction solutions before and after aging were dispersed in water, and their particle size distributions were investigated by dynamic light scattering (DLS). **Hep-Cyt*n*** (*n* = 0, 2, and 8) without aging, i.e., as-precipitated heparin-immobilized CaP nanoparticles, were dispersed well in water and exhibited number-average hydrodynamic diameters of 100–200 nm (Figure 5a, left). The size distributions of these nanoparticles remained unchanged even after aging (Figure 5a, right), although the aged nanoparticles immobilized a larger content of heparin and cytochrome C than those without aging (Figure 3 and Figure 4a). For all the tested nanoparticles without aging, the hydrodynamic diameters measured by DLS were larger than the primary particle size observed by SEM (Figure 1a–c) and TEM (ca. 20–30 nm, Figure 2a). This might be due to the different observation conditions: DLS measurements were conducted underwater, whereas SEM and TEM observations were conducted under dry conditions. The possibility that several primary particles came together in water cannot be denied. As opposed to the heparin-immobilized CaP nanoparticles described above, **Cyt*n*** (*n* = 0, 2, and 8), i.e., heparin-free CaP nanoparticles, with and without aging sedimented within an hour after dispersion in water; therefore, reliable DLS data were unavailable. These results suggested that heparin can act as a dispersant when co-immobilized with cytochrome C within CaP nanoparticles, as reported previously [14,16,17].

Heparin immobilized within the CaP nanoparticles provided them with relatively large negative zeta potentials, thereby improving the particle dispersibility. The zeta potentials of **Hep-Cyt*n*** (*n* = 0, 2, and 8) with and without aging ranged from −22 mV to −15 mV (Figure 5b). The large negative zeta potentials of the nanoparticles are likely due to heparin, which is rich in negatively charged sulfo and carboxyl groups, on their surfaces, and should contribute to their dispersion through electrostatic repulsion. This is supported by the result that **Cyt*n*** (*n* = 0, 2, and 8) with and without aging showed positive zeta potentials from +4 to +8 mV. The zeta potential absolute values of **Hep-Cyt8** were smaller (less negative) than those of **Hep-Cyt0** and **Hep-Cyt2** (Figure 5b), with or without aging. This is probably due to the charge cancellation by higher contents of basic protein, cytochrome C, in the former than in the latter two (Figure S2a). The latter nanoparticles with lower cytochrome C contents (**Hep-Cyt0** and **Hep-Cyt2**) had increased zeta potential absolute values (became more negative) after aging. This might be because of the increased heparin content on the surfaces of the nanoparticles after aging (Figure 4a).

### 2.6. Immobilization of Other Proteins

**Figure 6:**
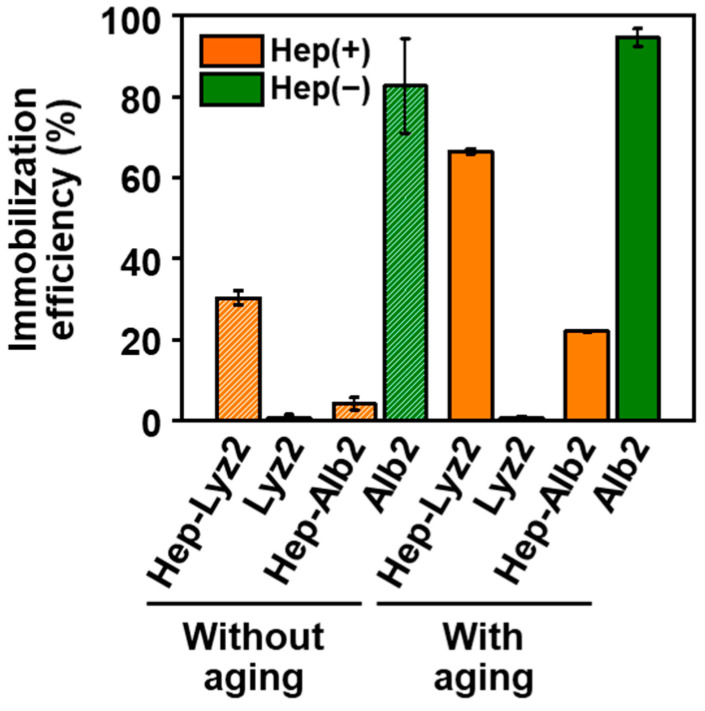
Immobilization efficiencies of lysozyme and albumin in the nanoparticles: **Hep-Lyz2**, **Lyz2**, **Hep-Alb2**, and **Alb2** with and without aging (average ± standard error, N = 2).

Heparin added to the reaction solution improved the immobilization efficiencies of another basic protein, lysozyme, within CaP nanoparticles, although it showed the opposite effect on an acidic protein, albumin. Here, lysozyme or albumin instead of cytochrome C was added to the reaction solutions with and without heparin. The products obtained in the presence of heparin are represented as **Hep-Lyz2** and **Hep-Alb2**, and the products obtained without the addition of heparin are represented as **Lyz2** and **Alb2**. The immobilization efficiencies of lysozyme in **Hep-Lyz2** with and without aging were 67% and 30%, respectively, while those in **Lyz2** with and without aging were both below 1% (Figure 6). This result indicated that lysozyme was immobilized within the nanoparticles in the presence of heparin in the reaction solution, as in the case of cytochrome C, although it was hardly immobilized in the absence of heparin even after aging. In contrast to lysozyme and cytochrome C, albumin was immobilized within the nanoparticles with lower efficiency in the presence of heparin than in the absence of heparin in the reaction solution with or without aging (Figure 6). The immobilization efficiencies of albumin in **Alb2** with and without aging were as high as 95% and 83%, respectively, while those in **Hep-Alb2** with and without aging were 22% and 4%, respectively.

Heparin immobilized within the nanoparticles provided them with relatively large negative zeta potentials and allowed their dispersion in water, regardless of the type and content of the protein (lysozyme and albumin). Both **Hep-Lyz2** and **Hep-Alb2** with and without aging showed large negative zeta potential values (from −21 mV to −17 mV) and were well-dispersed in water (Figure S5), most likely due to heparin being immobilized within the nanoparticles. In contrast, the zeta potential absolute values of **Lyz2** and **Alb2** (free of heparin) with and without aging were relatively small (from −4 mV to +6 mV). Consequently, all of them were sedimented within an hour after dispersion in water (reliable DLS data were unavailable). These results reconfirmed the critical role of heparin as a dispersant in the nanoparticles.

## 3. Discussion

The putative formation mechanism of heparin-immobilized CaP nanoparticles (**Hep-Cyt0**) in the heparin-containing reaction solution (without any other proteins) is as follows. First, homogeneous nucleation of CaP (amorphous phase) occurred throughout the reaction solution to form spherical CaP nanoparticles with a primary particle size of about 20–30 nm during initial mixing for 1 min (Figure 1a and Figure 2a). In this stage, heparin was immobilized within the CaP nanoparticles through electrostatic interactions between calcium ions in CaP and sulfo and carboxyl groups in heparin [14,16,17]. The heparin-immobilized CaP nanoparticles dispersed well in water owing to their large negative zeta potential (−17 mV, Figure 5b). This is attributed to the negatively charged functional groups in heparin present on the surfaces of nanoparticles. During subsequent aging for 24 h, the spherical nanoparticles underwent physicochemical changes: they deformed into irregularly shaped nanoparticles (Figure 1d and Figure 2d) with an increased Ca/P elemental ratio (from 1.52 to 1.67, Figure 4b). These changes were derived from the crystallization of isotropic amorphous CaP into anisotropic apatite crystals (Figure 2c,f). Such an amorphous-to-crystalline transformation of CaP is a well-known spontaneous reaction under a supersaturated environment [20]. In the meantime, the nanoparticles further immobilized heparin on their surfaces during aging, leading to increases in their S/Ca elemental ratio (from 0.08 to 0.12, Figure 4a) and zeta potential absolute value (from −17 mV to −22 mV, Figure 5b). Despite these changes, the nanoparticles showed no significant changes in size distribution (Figure 5a) or the contents of Ca and P (Figure S4a,b) due to aging. This suggests that CaP precipitation from the reaction solution was almost finished in the initial mixing step for 1 min, and the precipitated CaP underwent maturation during subsequent aging.

When cytochrome C and heparin coexisted in the reaction solution, cytochrome C as well as heparin were immobilized within the CaP nanoparticles. A certain amount of cytochrome C was immobilized within the CaP nanoparticles during initial mixing for 1 min even in the absence of heparin in the reaction solution, as indicated by the immobilization efficiencies of cytochrome C in **Cyt2** (12%) and **Cyt8** (8%) without aging (Figure 3). Heparin in the reaction solution enhanced the immobilization of cytochrome C within the nanoparticles, resulting in higher immobilization efficiencies of cytochrome C in **Hep-Cyt2** (39%) and **Hep-Cyt8** (25%) without aging (Figure 3). During subsequent aging for 24 h, cytochrome C, as well as heparin, were further immobilized on the nanoparticles, increasing the contents of cytochrome C (Figure 3) and heparin (Figure 4a). After aging, the immobilization efficiency of cytochrome C reached as high as 76% in **Hep-Cyt2** and 52% in **Hep-Cyt8** (Figure 3). The co-immobilization of heparin and cytochrome C within CaP nanoparticles should be due to the electrostatic interactions between the basic protein cytochrome C and heparin, which has high negative charge densities [18,19]. This is evident from the results of the comparative experiments; heparin in the reaction solution promoted immobilization of another basic protein, lysozyme, within the nanoparticles, although it exhibited the opposite effect on the acidic protein, albumin (Figure 6). These results correspond to a previous report by Kojima et al. in which cytochrome C adsorbed better on negatively charged apatite nanoparticles modified with poly(L-glutamic acid) compared to positively charged apatite nanoparticles modified with poly(L-lysine) [21].

The present study clarified that heparin-immobilized CaP nanoparticles have the capacity to efficiently co-immobilize basic proteins rather than acidic proteins in the present precipitation process. There are many clinically available basic proteins, such as cytokines and antibodies, whose delivery to cells and tissues at target sites in the body can be valuable. Therefore, the findings in this study will be useful as a design guide for the fabrication of CaP-based nanoparticles for protein delivery applications.

## 4. Materials and Methods

### 4.1. Preparation of Nanoparticles Immobilizing Heparin and/or Cytochrome C

We prepared six types of reaction solutions containing calcium ions, phosphate ions, sodium carbonate, heparin, and various concentrations of cytochrome C. Calcium Chloride Corrective Injection 1 mEq/mL (Otsuka Pharmaceutical Co., Ltd., Tokyo, Japan) and Dibasic Potassium Phosphate Injection 20 mEq Kit (Terumo Corporation, Tokyo, Japan) were used as calcium and phosphate ion solutions (500 mM), respectively. A sodium carbonate solution (500 mM) was prepared by adding sodium carbonate (FUJIFILM Wako Pure Chemical Corporation, Osaka, Japan) to ultrapure water in a glass vial, followed by sonicating for a few minutes until complete dissolution. A heparin solution (5 mg/mL) was prepared by dissolving heparin sodium (FUJIFILM Wako Pure Chemical Corporation) in saline solution (OTSUKA NORMAL SALINE, Otsuka Pharmaceutical Co., Ltd.) by the same procedure. Before preparing a reaction solution, three solutions (solutions A, B, and C) were prepared. Solution A was prepared by mixing (vortexed for a few minutes) the calcium ion solution (500 mM), heparin solution (5 mg/mL), and saline solution at volume ratios of 8:25:17 in a centrifuge tube (with a capacity of 50 mL). Solution B was prepared by mixing (vortexed for a few minutes) the phosphate ion solution (500 mM), sodium carbonate solution (500 mM), and saline solution at volume ratios of 4:4:17 in a centrifuge tube (with a capacity of 50 mL). Solution C was prepared by adding cytochrome C (NACALAI TESQUE Inc., Kyoto, Japan) to the saline solution in a glass vial at concentrations of 0, 1, 2, 3, 5, and 8 mg/mL, followed by sonicating for a few minutes until complete dissolution. Here, we referred to a report by Liang et al. [14] for the composition of solutions A and B. The prepared solutions A, B, and C were kept in a dry bath with a setting temperature of 25 °C. The reaction solution was prepared by adding solution B (1 mL), solution C (1 mL), and solution A (2 mL) in the order listed above quickly to a centrifuge tube (with a capacity of 15 mL) in the dry bath at 25 °C by using pipettes. Immediately after the addition of solution A, the reaction solution (4 mL) was vortexed for 1 min. Products were collected from the reaction solution immediately after mixing (without aging) or after subsequent aging for 24 h. The product without aging was collected immediately after mixing via centrifugation (6000 rpm, 5 min) followed by washing three times with ultrapure water. In the aging process, the tube (just after mixing) was tightly sealed and then shaken at 150 rpm in a thermostatic shaker (M·BR-104P, TAITEC CORPORATION, Koshigaya, Japan) at a setting temperature of 25 °C. After shaking (aging) for 24 h, the resulting precipitate was collected and washed three times with ultrapure water. The products obtained with and without aging are represented as **Hep-Cyt*n*** (*n* = 0, 1, 2, 3, 5, and 8) according to the concentration of cytochrome C (*n* mg/mL) in solution C. The products excluding heparin were also prepared in a similar manner without the addition of heparin to solution A, and are represented as **Cyt*n*** (*n* = 0, 2, and 8).

### 4.2. Characterization of the Products

The products were mounted on a silicon substrate and dried under reduced pressure. The morphology of the products was examined using SEM (S-4800, Hitachi High-Tech Corporation, Tokyo, Japan) after sputter coating with gold using a sputter coating machine (SC-701MkII, Sanyu Electron Co., Ltd., Tokyo, Japan). The chemical composition of the products was examined without coating using EDX (AZtecOne, Oxford Instruments plc, Abingdon, UK) equipped in a tabletop SEM (TM4000Plus II, Hitachi High-Tech Corporation).

The nano- and crystalline structures of the selected products (**Hep-Cyt0** and **Hep-Cyt8**) were further investigated using TEM (Tecnai Osiris, FEI, Hillsboro, OR, USA) operated at 200 kV. The products were mounted on a holey carbon film on a copper grid and dried under reduced pressure prior to analysis. The crystalline structures of the products were examined by SAED analysis.

The contents of Ca, P, and S in the products obtained from a single batch of each reaction solution (4 mL) were determined by chemical analysis as follows. The products were dispersed in ultrapure water (10 mL) by sonication. To this dispersion (1 mL), 5 mL of 6 M HCl solution (FUJIFILM Wako Pure Chemical Corporation) and 4 mL of ultrapure water were added. After complete dissolution of the products and secondary dilution (only for Ca and P, 20 times) with 0.6 M HCl solution, Ca, P, and S in the HCl solutions were quantified using ICP-OES (ULTIMA2, HORIBA, Ltd., Kyoto, Japan). Two independent batches were used to obtain the average and standard error values.

The immobilization efficiencies of cytochrome C in the products were calculated by dividing the contents of cytochrome C in the products by the total amounts of cytochrome C in the reaction solutions. The contents of cytochrome C in the products were determined as follows. After the first centrifugation following mixing or aging (Section 4.1), the supernatant was removed from the tube and diluted 3 or 8 times with saline solution. The amount of cytochrome C in the diluted supernatant solution was analyzed by the absorbance at 409 nm using a UV–visible spectrophotometer (UV-2450, SHIMADZU CORPORATION, Kyoto, Japan). The contents of cytochrome C in the products were calculated by subtracting the amounts of residual cytochrome C in the supernatant from the total amounts of cytochrome C in the reaction solutions. Two (for **Cyt2** and **Cyt8** with aging) or three (for others) independent batches were used to obtain the average and standard error values.

### 4.3. Size Distribution and Zeta Potential of the Products

The size (hydrodynamic diameter) distributions and zeta potentials of the products dispersed in ultrapure water were measured at 20 °C through DLS and electrophoretic light scattering (ELS) with a particle size analyzer (ZETASIZER Nano-ZS, Malvern Instruments Ltd., Worcestershire, UK). Before DLS and ELS measurements, the products obtained from a single batch of each reaction solution (4 mL) were dispersed in ultrapure water (10 mL) by sonication. The measurements were repeated three times per dispersion to obtain the average and standard deviation of the measured values.

### 4.4. Preparation of Nanoparticles Immobilizing Heparin and/or Lysozyme or Albumin

The products immobilizing lysozyme or albumin were obtained in the same procedure as above (Section 4.1), except that lysozyme (FUJIFILM Wako Pure Chemical Corporation) or albumin (FUJIFILM Wako Pure Chemical Corporation) were dissolved in the saline solution for preparing solution C instead of cytochrome C. The concentration of lysozyme or albumin in solution C was fixed at 2 mg/mL. The lysozyme- and albumin-immobilized products are represented as **Hep-Lyz2** and **Hep-Alb2**, respectively. The products excluding heparin were also prepared in a similar manner without the addition of heparin to solution A and are represented as **Lyz2** and **Alb2**.

The immobilization efficiencies of lysozyme and albumin in the products were calculated in a similar manner as described in Section 4.2. The absorbances at 281 nm and 278 nm were measured to analyze the amounts of lysozyme and albumin, respectively, in the diluted supernatant solution. Two independent batches were used to obtain the average and standard error values.

## 5. Conclusions

CaP nanoparticles co-immobilizing heparin and cytochrome C, a model basic protein, were obtained by a precipitation process in a reaction solution containing calcium ions, phosphate ions, heparin, and cytochrome C. The immobilized heparin acted as a dispersant; it provided a large negative zeta potential to the nanoparticles and enabled their dispersion in water. More importantly, heparin acted as an immobilizing agent for cytochrome C; it remarkably improved the immobilization efficiency of cytochrome C within the nanoparticles. The content of cytochrome C in the nanoparticles was increased by aging the nanoparticles and by increasing the cytochrome C concentration in the reaction solution. Heparin showed a similar immobilizing effect on another basic protein, lysozyme, although it showed the opposite effect on the acidic protein, albumin. The heparin-immobilized CaP nanoparticles have a greater immobilizing capacity for basic proteins than for acidic proteins, which makes them useful as a carrier for basic protein delivery.

## Data Availability

Not applicable.

## Supplementary Materials

The following supporting information can be downloaded at: https://www.mdpi.com/article/10.3390/ijms231911530/s1.
