# Supplementary material for: High Immobilization Efficiency of Basic Protein within Heparin-Immobilized Calcium Phosphate Nanoparticles
Source: Int J Mol Sci. 2022 Sep 29;23(19):11530. doi: 10.3390/ijms231911530 (PMC9569611; doi:10.3390/ijms231911530)
Supplement: Supplementary file 1 [file ijms-23-11530-s001.zip › ijms-1931094-supplementary.pdf]

High Immobilization Efficiency of Basic Protein within Heparin-immobilized Calcium Phosphate Nanoparticles

Maki Nakamura [1,*], Wakako Bunryo [1], Aiko Narazaki [2] and Ayako Oyane [1]

[1] *Nanomaterials Research Institute, National Institute of Advanced Industrial Science and Technology (AIST), Central 5, 1-1-1 Higashi, Tsukuba, Ibaraki 305-8565, Japan.*

[2] *Research Institute for Advanced Electronics and Photonics, National Institute of Advanced Industrial Science and Technology (AIST), Central 2, 1-1-1 Umezono, Tsukuba, Ibaraki 305-8568, Japan.*

*Corresponding author: Maki Nakamura

E-mail: ma-ki-nakamura@aist.go.jp; Tel: +81-29-861-4604

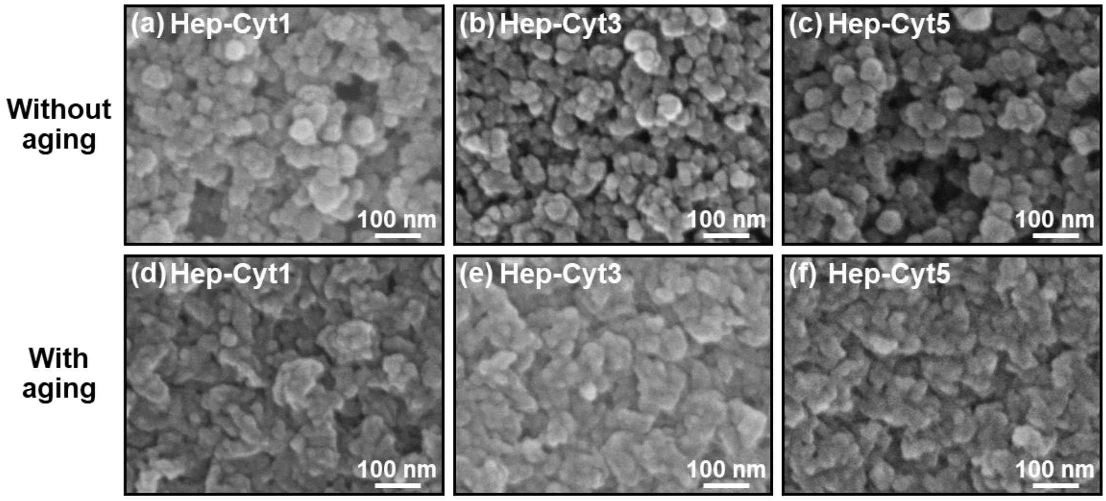

Figure S1. SEM images of the products: **Hep-Cyt1**, **Hep-Cyt3**, and **Hep-Cyt5** with (d–f) and without (a–c) aging.

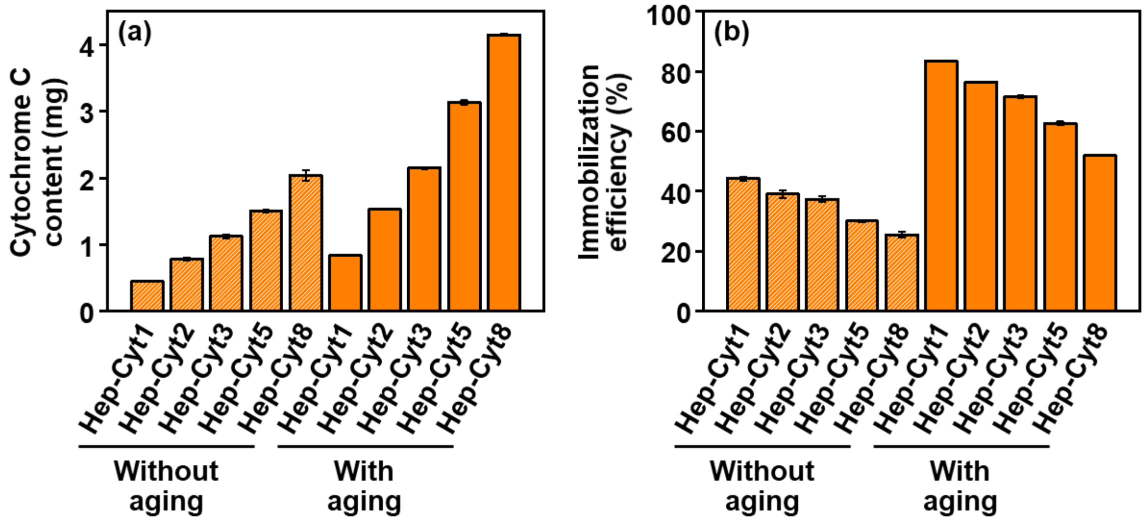

Figure S2. Contents (a) and immobilization efficiencies (b) of cytochrome C in the nanoparticles: **Hep-Cyt1**, **Hep-Cyt2**, **Hep-Cyt3**, **Hep-Cyt5**, and **Hep-Cyt8** with and without aging (average ± standard error, N = 3).

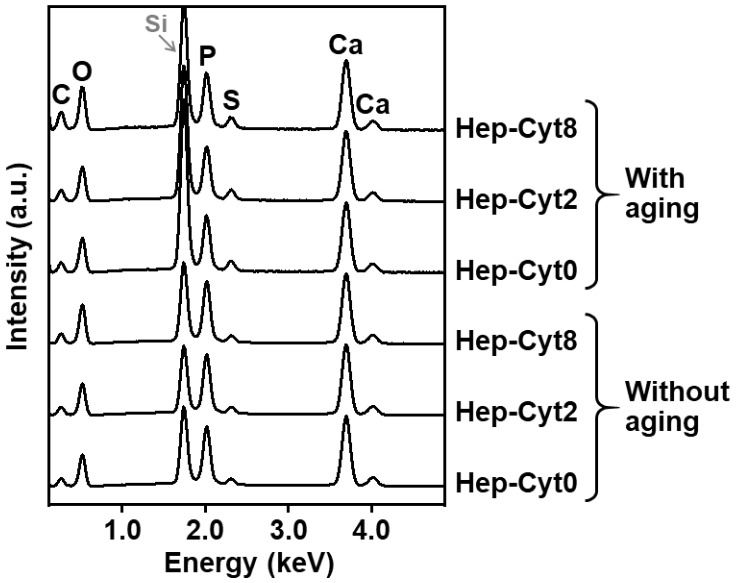

Figure S3. EDX spectra of the nanoparticles: **Hep-Cyt0**, **Hep-Cyt2**, and **Hep-Cyt8** with and without aging. The peak for Si was attributed to the silicon substrate used for mounting the nanoparticles.

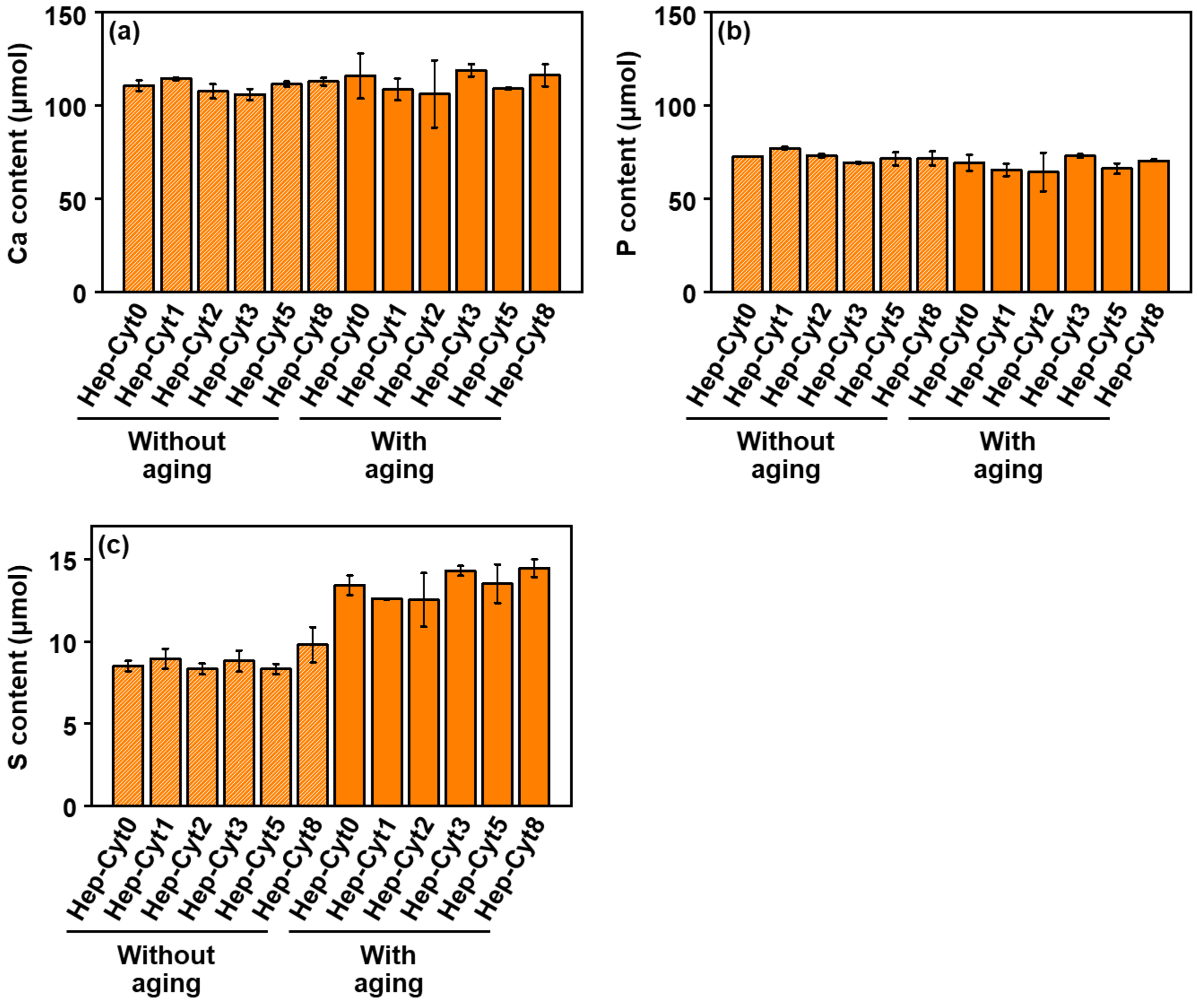

Figure S4. Contents of Ca (a), P (b), and S (c) in the nanoparticles: **Hep-Cyt0**, **Hep-Cyt1**, **Hep-Cyt2**, **Hep-Cyt3**, **Hep-Cyt5**, and **Hep-Cyt8** with and without aging (average ± standard error, N = 2).

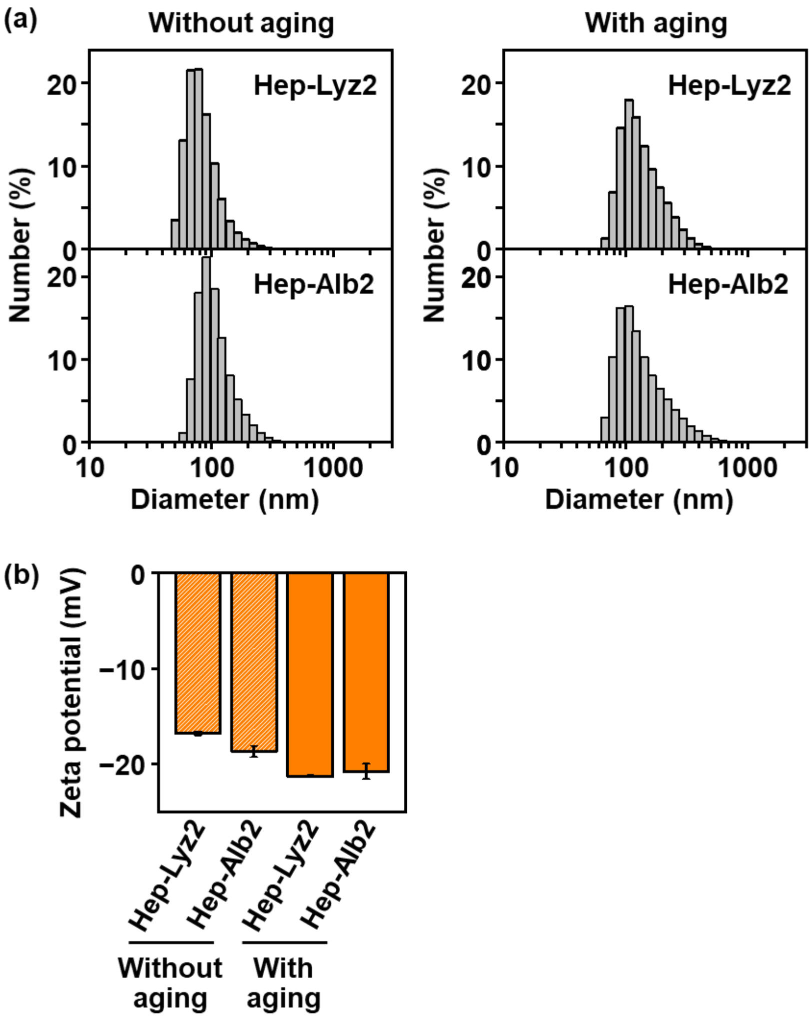

Figure S5. DLS histograms of number distributions (a) and zeta potentials (b) of the nanoparticles: **Hep-Lyz2** and **Hep-Alb2** with and without aging. The nanoparticles were dispersed in water.
